# Practical Approaches to the Use of Lenalidomide in Multiple Myeloma: A Canadian Consensus

**DOI:** 10.1155/2012/621958

**Published:** 2012-10-14

**Authors:** Donna Reece, C. Tom Kouroukis, Richard LeBlanc, Michael Sebag, Kevin Song, John Ashkenas

**Affiliations:** ^1^Princess Margaret Hospital, University Health Network, 610 University Avenue, Toronto, ON, Canada M5G 2M9; ^2^Department of Oncology, Juravinski Cancer Centre, 699 Concession Street, Hamilton, ON, Canada L8V 5C2; ^3^Hôpital Maisonneuve-Rosemont, University of Montreal, Montreal, QC, Canada H1T 2M4; ^4^McGill University Health Centre, McGill University, Montreal, QC, Canada H3A 1A1; ^5^Leukemia/BMT Program of British Columbia, Vancouver General Hospital, Vancouver, BC, Canada V5Z 1M9; ^6^SCRIPT, Toronto, ON, Canada M4S 1Z9

## Abstract

In Canada, lenalidomide combined with dexamethasone (Len/Dex) is approved for use in relapsed or refractory multiple myeloma (RRMM). Our expert panel sought to provide an up-to-date practical guide on the use of lenalidomide in the managing RRMM within the Canadian clinical setting, including management of common adverse events (AEs). The panel concluded that safe, effective administration of Len/Dex treatment involves the following steps: (1) lenalidomide dose adjustment based on creatinine clearance and the extent of neutropenia or thrombocytopenia, (2) dexamethasone administered at 20–40 mg/week, and (3) continuation of treatment until disease progression or until toxicity persists despite dose reduction. Based on available evidence, the following precautions should reduce the risk of common Len/Dex AEs: (1) all patients treated with Len/Dex should receive thromboprophylaxis, (2) erythropoiesis-stimulating agents (ESAs) should be used cautiously, and (3) females of child-bearing potential and males in contact with such females must use multiple contraception methods. Finally, while Len/Dex can be administered irrespective of prior therapy and in all prognostic subsets, patients with chromosomal deletion 17(p13) have less favorable outcomes with all treatments, including Len/Dex. New directions for the use of lenalidomide in RRMM are also considered.

## 1. Introduction

Multiple myeloma (MM), the second most common hematological malignancy in adults, is associated with various clinical manifestations including anemia, lytic bone lesions, and renal and immune impairments. According to Canadian Cancer statistics, an estimated 2300 Canadians will be diagnosed with MM and 1350 will die from this disease in 2011 [1]. While no cure for MM is available, five-year survival rates have risen substantially in Canada and elsewhere over the last decade, partly due to novel therapies such as thalidomide, bortezomib, and lenalidomide [2, 3]. Nonetheless, regardless of initial treatment, most patients will eventually relapse and require salvage therapy, often consisting of novel agents, alone or in combination.

Lenalidomide is an immunomodulatory drug with direct effects on myeloma cells as well as their microenvironment. Early clinical trials with lenalidomide as a single agent in relapsed or refractory MM (RRMM) patients demonstrated its antimyeloma activity [4]. In preclinical studies, the agent has been shown to kill myeloma cells by upregulating certain cyclin-dependent kinase inhibitors and other early response factors [5]. Lenalidomide can also induce apoptosis by the activation of the intrinsic caspase-8 pathway [6], and it is thought to be more potent than thalidomide at inhibiting MM cell line growth and inhibiting TNF-**α** secretion from peripheral blood cells following LPS stimulation [7, 8]. Lenalidomide also has antiangiogenic properties, manifested *in vitro* by its ability to inhibit endothelial cell migration [9]. In addition, lenalidomide has properties not shared by thalidomide, such as inhibition of T regulatory cells and enhancement of tumor immunity [10, 11].

As reported in two landmark phase III trials that are the basis of Canadian approval of lenalidomide in RRMM, the efficacy of this agent is greatest when used in combination with dexamethasone [12, 13]. This combination is supported by data showing that lenalidomide can activate caspases 3, 8, and 9 with variable efficiency in different MM cell lines and that the addition of dexamethasone is synergistic and leads to a greater induction of apoptosis [5]. 

Additional studies, subgroup analyses of available phase III trials, and Canadian postmarketing experiences have all informed current practice regarding the use of lenalidomide in the RRMM patient population. In this paper we aim to provide an up-to-date practical guide on the use of this novel agent in the setting of RRMM, as well as a guide to managing commonly seen adverse events. To the best of our knowledge, the current report provides the first Canadian guidance for using lenalidomide in RRMM.

## 2. Methods

The expert panel convened in Paris, France, on May 2, 2011, in conjunction with the 13th International Myeloma Workshop. The group met to discuss the use of lenalidomide in the management of RRMM in the Canadian environment. The Chair (DR) invited panelists to research and write individual sections of the paper.

The various sections were collected, compiled, and distributed to the group, which discussed the paper via web conference. Panelists subsequently generated a revised draft in which all sections included specific clinical guidance (i.e., practice considerations). The revised paper was discussed at a final web conference, where all practice recommendations were considered, revised as appropriate, and ultimately adopted by the full panel; any areas of disagreement are noted.

Celgene Canada provided the impetus for the panel to pursue this project freely and independently. Celgene Canada supported the process throughout, including support for the participation of a medical writer (JA) in preparing this paper. The opinions represented here are solely those of the physician-panelists.

## 3. Indication, Timing, Dose, and Treatment Duration

In October 2008, the combination of lenalidomide and dexamethasone (Len/Dex) was approved in Canada for the treatment of RRMM in patients who had received at least one prior therapy. This approval was based on evidence from two phase III trials, namely, MM009 [12] and MM010 [13], which showed significant benefits in response rate (RR), time to progression (TTP), and overall survival (OS) following Len/Dex therapy, compared with dexamethasone monotherapy. The benefits of Len/Dex over dexamethasone alone were seen in all age groups and were independent of previous therapy type. Based on the currently approved indication for this agent in Canada and the results of available studies, the initiation of lenalidomide therapy is not limited by the number or type of previous lines of therapy, although OS and progression-free survival are greater among patients with only one prior therapy versus those with two or more prior therapies [35].

In the MM009 and MM010 trials, lenalidomide was given at a dose of 25 mg per day on days 1 to 21 of a 28-day cycle, along with 40 mg of dexamethasone per day on days 1–4, 9–12, and 17–20. After the fourth cycle, 40 mg of dexamethasone was given daily on days 1–4 of every cycle. Cycles were continued until disease progression or until toxicity persisted, despite dose reduction. Noting the lack of prospective randomized trials specifically addressing different approaches to drug administration in the relapsed/refractory setting, the panel agreed that the recommended dose and schedule of Len/Dex therapy need not directly follow those outlined in published clinical trials.

We agreed that the starting dose of lenalidomide should remain at the current standard (25 mg daily on days 1–21) in the absence of baseline renal insufficiency and/or significant cytopenias. Specifically, the dose of lenalidomide must be adjusted based on the creatinine clearance, using standard dose adjustments (Table 1). Either the Cockcroft-Gault or the MDRD (modification of diet in renal disease) formula may be used to calculate creatinine clearance. Caution is urged in calculating the renal function based solely on serum creatinine level in older patients with MM [36]. 

Lenalidomide treatment should be used with caution in patients with thrombocytopenia (i.e., platelet counts <50 × 10^9^/L or <30 × 10^9^/L in those with heavy marrow infiltration with myeloma) and absolute neutrophil counts <1.0 × 10^9^/L; if lenalidomide is used in this setting, measures for aggressive growth factor supplementation and/or platelet transfusion support must be in place.

Although the pivotal phase III trials in RRMM used the standard high-dose (HD) pulsed dexamethasone (12 doses of 40 mg per month, on days 1–4, 9–12, and 17–20 of a 28-day schedule), it has become common practice in Canada to administer dexamethasone on a weekly schedule (four doses of 20–40 mg per month, on days 1, 8, 15, and 22 of a 28-day schedule). Although dexamethasone dose should be selected on the basis of individual clinical circumstances, the panel notes that such low-dose (LD) dexamethasone administration is particularly suitable for elderly patients, as well as those with uncontrolled diabetes, unmanageable glucocorticoid side effects, or relatively indolent relapses.

Weekly LD dexamethasone now represents the standard of care in newly diagnosed individuals. The panel's preference for LD dexamethasone administration is based in part on the results of a trial on Len/Dex in initial therapy for MM (see New Directions, below) [30]. Here, despite a somewhat lower RR compared to the HD dexamethasone group, patients receiving LD dexamethasone plus lenalidomide experienced improved OS and fewer grade ≥3 toxicities. A second line of evidence supporting the use of dexamethasone at doses lower than employed in MM009 and MM010 comes from a post hoc analysis of stepwise dose reduction in these trials [37]. In this analysis, which did not directly test a weekly dexamethasone regimen, patients who reduced the dose of dexamethasone experienced significantly better RR, TTP, and OS. A final reason for the use of weekly LD dexamethasone is more hypothetical: one purported important mechanism of action of lenalidomide is via its immunomodulatory properties, and laboratory and clinical studies have demonstrated that dexamethasone can antagonize the potentially beneficial immunostimulatory effects of this drug [5, 38]. Therefore, LD dexamethasone may allow better immunomodulatory effects, while preserving the ability of corticosteroids to enhance the antiproliferative activities of lenalidomide.

Finally, with regard to treatment duration, lenalidomide therapy should be maintained continuously in most patients. This is in contrast to regimens in which combination therapy is given to maximal response and then discontinued to allow a treatment hiatus. One study has reported that the duration of lenalidomide therapy is directly related to a longer survival [39]. However, the optimal dose of lenalidomide when administered on a long-term basis is less certain. For example, another post hoc analysis of the MM009/MM010 study examined patients who were still on therapy 12 months after entering the trial and found that those who had dose reductions after 12 months had a significantly longer PFS than those who had reductions less than 12 months earlier, or no dose reduction [40].

The panel makes no specific recommendation on routine dose reduction after a specific period of time. However, we recommend that doses of lenalidomide and/or dexamethasone should be reduced to allow treatment to continue until disease progression occurs. Dose interruptions should occur only in situations of significant toxicities, with a plan to reinstitute therapy as soon as toxicity decreases with appropriate dose modifications. If required, dose reduction to ameliorate toxicity should follow the recommendations outlined in Tables 1, 2, 3, and 4.

Practice considerations are as follows.

- Len/Dex is approved for the treatment of RRMM in patients who have received ≥1 prior therapy and is appropriate irrespective of the number or type of therapies previously given.
- Lenalidomide dose must be adjusted based on creatinine clearance.
- Dosing should take into account pre-existing and developing cytopenias.
- Dexamethasone is usually administered at doses of 20–40 mg once per week. However, this LD regimen has not been formally studied in the setting of relapsed myeloma, and the results may not be the same as those reported in the pivotal MM009/MM010 trials.
- Len/Dex treatment should be maintained as in the pivotal trials, that is, continued until disease progression or until significant toxicity persists despite dose reduction.

## 4. Treatment of Special Populations

### 4.1. High-Risk Multiple Myeloma

The definition of high-risk myeloma has evolved considerably over the past decade from one that predominantly relied on clinical and biochemical parameters (Durie-Salmon and ISS (International Staging System) stage, serum LDH (lactate dehydrogenase), CRP (C-reactive protein), proliferating index, etc.) to one that accounts for disease-specific cytogenetic and genomic factors. Several recurrent chromosomal aberrations—including chromosomal deletions (del(13q14), del(17p13)), translocations (t(4; 14), t(14; 16), t(14; 20)), and amplifications (1q21), as well as numerical chromosomal abnormalities (hypodiploid versus hyperdiploid karyotype)—correlate with poor disease outcomes. Similarly, genomewide gene expression profiling (GEP) studies have identified myeloma molecular subgroups with unique gene signatures that correlate with disease outcomes. In particular, a 70-gene signature was validated as a predictor of response to therapy and disease survival independently of clinical parameters and structural or numerical chromosomal abnormalities. Although most Canadian centers perform FISH cytogenetics for detection of del(13q14), del(17p13), and t(4; 14), genomic analyses are not routinely obtained.

To date, four retrospective studies have assessed the impact of cytogenetic abnormalities on outcomes of Len/Dex treatment among RRMM patients [15–18], as summarized in Table 5. The most consistent finding among these studies is that patients with del(17p13) experience less favorable outcomes when treated with Len/Dex [15, 16] or Len/Dex with bortezomib than those individuals lacking this adverse prognostic factor [18]. However, the presence of del(17p13) has repeatedly been shown to predict a shorter progression-free survival (PFS) and OS among RRMM patients, regardless of therapy [15, 16, 18]. Although patients with del(17p13) derive less benefit, the panel agreed that they may be treated with Len/Dex but should preferentially be considered for clinical trials designed for high-risk patients, if such an option is available. Innovative strategies, not yet defined, are needed for patients with a 17p13 deletion.

Although the trials of Reece et al. [15] and Klein et al. [16] suggest that Len/Dex treatment can overcome the poorer prognosis ordinarily associated with del(13q14) and t(4; 14), these conclusions are in contrast to those of Avet-Loiseau et al. [17]. In this last study, del(13q14) and t(4; 14) were associated with significantly lower RR, PFS, and OS in univariate analysis. In particular, patients with t(4; 14), compared to patients without t(4; 14), experienced significantly lower response and survival rates. However, multivariate regression analysis identified a prior history of progression while on thalidomide as the main adverse prognostic factor, and t(4; 14) per se was not retained in the model. Moreover, the patients in the Avet-Loiseau trial were more heavily pretreated. Evidence to date is also equivocal regarding the impact on Len/Dex treatment efficacy of chromosome 1q21 amplifications [16, 18].

With regard to high-risk myeloma, as defined by the 70-gene GEP signature, there are currently no studies assessing the impact of lenalidomide-based therapy on the survival of these patients when used in the relapsed setting. However, results of studies incorporating lenalidomide in the frontline treatment regimen (e.g., Total Therapy 3, incorporating multidrug induction therapy, tandem autologous stem cell transplantation, and maintenance with the combination of lenalidomide, bortezomib, and dexamethasone) suggest that the 70-gene GEP signature remains a predictor of poor survival outcomes [41].

Currently, it remains difficult to provide definite recommendations for the use of lenalidomide in relapsed patients with high-risk cytogenetics. Prospective studies in this area are clearly warranted.

Practice considerations are as follows.

- Based on the results of a Canadian analysis of the Expanded Access Program of Len/Dex in relapsed/refractory myeloma patients, Len/Dex may be effective in patients with t(4; 14) or del(13q14) identified by FISH (fluorescence in situ hybridization) cytogenetics.
- Patients with del17(p13) have poorer outcomes with all treatments, including Len/Dex treatment, and are high-priority candidates for innovative regimens directed to high-risk patients. However, Len/Dex may be used in the absence of such alternatives.

### 4.2. Previous Thalidomide Treatment

Although lenalidomide has been shown to be more potent than thalidomide in preclinical studies, the two agents are structurally similar and likely exert their antimyeloma effects through similar mechanisms. Retrospective investigations suggest that prior thalidomide exposure [15], progression during thalidomide [17], and thalidomide resistance [18] independently predict reduced PFS and OS.

The MM-009 and MM-010 phase III studies included 154 (44%) and 120 (34%) patients, respectively, who had been previously exposed to thalidomide [12, 13]. A post hoc analysis of these two studies demonstrated that, while the overall RR of lenalidomide treatment was lower in patients previously treated with thalidomide (65% versus 54%), the response duration was not statistically different [19]. Further subgroup analyses of patients with prior thalidomide exposure revealed that those who had responded to thalidomide and did not progress while on therapy had the best overall RR, median duration of response, and PFS when subsequently treated with lenalidomide (Table 6). RR and PFS among patients who failed to respond to thalidomide were better with Len/Dex than with dexamethasone alone, although duration of response to the assigned agent did not differ. Finally, PFS was superior with Len/Dex over dexamethasone monotherapy, regardless of prior thalidomide response. Another nonrandomized, prospective study of 106 previously thalidomide-treated patients suggested that the overall RR, PFS, and OS were not significantly different between patients who were thalidomide-sensitive versus thalidomide-resistant (56%, 10 months, 17 months, resp.) [42]. A third study, retrospective in nature, looked at retreatment with immunomodulatory agents in patients given this class of drugs as initial therapy for myeloma. For the subset of patients who received Len/Dex after initial thalidomide, the overall RR was 48% and the median TTP was 9 months [43].

Practice considerations are as follows.

- Although treatment efficacy may be somewhat reduced, Len/Dex is an appropriate treatment choice among patients previously treated with thalidomide, irrespective of their earlier response.

### 4.3. Elderly Patients

Up to 37% of newly diagnosed MM patients are older than 75 years [44]. Elderly patients are more likely to have significant comorbidities, tend to be frail, and have lower performance status and poorer tolerance to medications. Nevertheless, elderly patients have often been included in clinical studies of novel agents, and available evidence suggests that, with appropriate management, they can also benefit from these agents. However, a 40 mg dose of dexamethasone can be challenging to deliver to some elderly patients, and this agent may be given at a lower weekly dose of 20 mg.

Practice considerations are as follows.

- Among elderly patients, dexamethasone should be started at a dose of 40 mg per week, unless there are significant and/or severe comorbidities.
- Dexamethasone should be started at a dose of 20 mg per week in less fit patients; an initial dose of 16 mg may be considered for very frail patients, as guided by clinical judgment. As noted above, these doses are lower than those used in the MM009/MM010 studies and the results may not be the same as when 4-day pulses are administered.

## 5. Toxicities and Management of Adverse Events

The safety and toxicity of lenalidomide have been evaluated in published clinical trials [12, 13], as well as in an expanded-access program for Canadian and international patients [45]. Although lenalidomide is well tolerated by most patients, some adverse effects are common during treatment. However, some of the more significant side effects associated with thalidomide are not seen with lenalidomide. Indeed, in the MM-009 and MM-010 studies, the incidences of grade 3-4 constipation, somnolence, and peripheral neuropathies were similar for the Len/Dex-treated group compared to the dexamethasone monotherapy group [12, 13]. Importantly, side effects associated with Len/Dex are not affected by the number of prior therapies [35].

### 5.1. Hematologic Toxicities

The most common grade 3-4 adverse events in the two phase III pivotal trials of lenalidomide were hematologic, including neutropenia; thrombocytopenia; to a lesser extent, anemia. The risk of grade 3 or 4 febrile neutropenia was slightly increased with the addition of lenalidomide (3.4% in the Len/Dex group versus 0% in the dexamethasone group). Dose reductions typically occur most frequently during the initial cycles. It is not clear whether the risks of neutropenia and thrombocytopenia per se decrease with time or whether the pattern observed is secondary to dose modifications [46, 47]. At any rate, clinicians should be particularly vigilant during the first few months after initiation of lenalidomide. Given that a standard lenalidomide dose of 25 mg among patients with renal failure is associated with more cytopenias, especially neutropenia and thrombocytopenia [48], reducing the initial dose may ameliorate these risks. Specific recommendations for laboratory monitoring are summarized below.

#### 5.1.1. Neutropenia and Thrombocytopenia

Myelosuppression associated with lenalidomide is dose-dependent and is usually predictable and manageable [47]. To decrease risks of infection and bleeding, lenalidomide should not be started in patients with an absolute neutrophil count (ANC) below 1.0 × 10^9^/L or a platelet count below 50 × 10^9^/L except in exceptional circumstances and with supportive measures in place, as discussed above. Lenalidomide administration should be interrupted whenever neutrophil and platelet counts reach these cutoffs. At the next cycle, if neutropenia is the only dose-limiting toxicity, treatment may resume at the same dose, with the addition of growth factor support such as filgrastim 300 or 480 mcg administered subcutaneously once or twice weekly, in patients with ANC >1.0 × 10^9^/L. In the presence of other dose-limiting toxicities, dose reduction is recommended (Table 2). Treatment may also be reintroduced, albeit at a reduced level, when platelet count is over 30 × 10^9^/L. For each subsequent grade 3-4 neutropenia and platelet count less than 30 × 10^9^/L, lenalidomide administration should be withheld and restarted at a lower dose at the next cycle. Dose adjustments for neutropenia and thrombocytopenia associated with lenalidomide are presented in Tables 3 and 4. In some circumstances, especially during the first few cycles, significant neutropenia or thrombocytopenia can result from heavy myeloma bone marrow infiltration rather than pure myelosuppression. In these cases, lenalidomide should probably be continued with the addition of G-CSF (granulocyte-colony stimulating factor) in case of neutropenia and platelet transfusions given to manage thrombocytopenia.

#### 5.1.2. Anemia

Anemia is rarely a significant problem in patients undergoing Len/Dex combination therapy. Thus, clinicians should follow the standard practice established by their institution for transfusions. Some concerns have been raised regarding the potential risk of venous thromboembolic events associated with concomitant use of erythropoietin. Although the MM-010 study [13] suggested that these events are unrelated, the MM-009 [12] study identified a trend toward more venous thromboembolic events with erythropoietin. Accordingly, we recommend that erythropoiesis-stimulating agents (ESAs) be used with caution in patients receiving lenalidomide; if an ESA is given, the hemoglobin level should be maintained at <120 g/L as per the Health Canada label.

#### 5.1.3. Others

Recently, lenalidomide exposure has been associated with failure to mobilize a sufficient number of stem cells using growth factors alone [49–51]. This negative effect on stem cell mobilization can be overcome with the addition of cyclophosphamide [52] or plerixafor [53]. Since use of lenalidomide most commonly follows relapse after autologous stem cell transplant (ASCT) and successful stem cell mobilization in eligible patients, this consideration is rarely problematic in Canada.

Practice considerations are as follows.

- MM patients experiencing neutropenia or thrombocytopenia should interrupt lenalidomide treatment until their ANC reaches 1.0 × 10^9^/L and/or their platelet count reaches 30 × 10^9^/L. Lenalidomide may then be restarted at a lower dose, as indicated in Table 2.
- The timing of interrupting and restarting lenalidomide in response to neutropenia and thrombocytopenia should follow the guidance in Tables 3 and 4, respectively.
- To avoid a potential increase in the risk of venous thromboembolism, ESAs should be utilized cautiously with Len/Dex, and the hemoglobin level should be maintained at <120 g/L.

### 5.2. Nonhematological Toxicities

Many nonhematological adverse effects reported with the combination of Len/Dex are associated with dexamethasone alone, including insomnia, peripheral edema, tremor, muscle weakness, blurred vision, dyspepsia, psychological changes, and hyperglycemia. These adverse events should be managed in the usual manner; if significant and persistent, they may necessitate dexamethasone dose reduction. Additionally, lenalidomide is potentially associated with gastrointestinal symptoms such as diarrhea, constipation, and nausea, as well as with muscle cramps, fatigue, and muscle weakness. As a general rule, for grade 3-4 nonhematological treatment-related toxicities, lenalidomide treatment should be withheld and restarted at the next lower dose level when toxicity has resolved to grade 2 or lower.

#### 5.2.1. Infections

Despite the immunomodulatory effect of lenalidomide, the infection rate was increased with the addition of lenalidomide in both the MM-009 and MM-010 trials [12, 13]. Most infections were low-grade, with grade 3-4 infections seen in 10–20% of patients. Per study protocol, no antibiotic prophylaxis was provided in either of the two phase III trials. Due to this risk of infection, antibiotic prophylaxis may be considered for patients treated with Len/Dex, especially if HD dexamethasone is used. Unfortunately, there currently exists no recommendation for a single antibiotic class for this purpose, but our own preference is levofloxacin. Given that use of LD dexamethasone is associated with less frequent infections in newly diagnosed patients [30], it is not clear whether routine antibiotic prophylaxis is necessary.

In the MM-009 and MM-010 studies, reports of grade 3-4 viral or fungal infections were rare [54].

Practice Considerations are as follows.

- LD dexamethasone is associated with a lower risk of infection than HD dexamethasone among new MM patients.
- Given the modest elevation in the risk of infection with Len/Dex treatment, antibiotic prophylaxis may be considered. Acceptable agents include trimethoprim/sulfamethoxazole or levaquin.

#### 5.2.2. Thromboembolic Events

Although the risk of thromboembolic events is low when lenalidomide is administered as a single agent [4], this risk increases when it is used in combination with dexamethasone. The incidences of thromboembolic events in the MM-009 and MM-010 studies were 8.8–14.7% with Len/Dex versus 3.4–4.7% with dexamethasone alone. However, thromboprophylaxis was not required in either of these studies.

The risk of venous thromboembolism is higher within the first few months after initiation of therapy with Len/Dex, decreasing dramatically thereafter [46]. This observation might be explained in part by the administration of higher doses of dexamethasone during the first 4cycles of therapy, followed by a significant decrease. Indeed, an Eastern Cooperative Oncology Group (ECOG) trial has shown that the incidence of thromboembolism is directly related to dexamethasone dose [30]. The risk of venous thromboembolism among MM patients treated with Len/Dex is comparable to that of other high-risk populations for whom thromboprophylaxis is commonly recommended. A number of prophylactic approaches have been suggested when immunomodulatory agents are administered, including those based on the number of potential risk factors for venous thromboembolism [55, 56]. However, a recently published phase III trial reported similar rates of thrombosis when either enoxaparin or ASA was used as thromboprophylaxis in transplant-eligible patients with newly diagnosed MM treated with lenalidomide-based regimens [57].

In the absence of randomized phase III trials comparing the thromboprophylaxis agents with a control/placebo group in an RRMM setting, it is difficult to draw conclusions concerning the real efficacy of these regimens. Nevertheless, the panel endorsed an approach in which daily ASA was suggested as thromboprophylaxis in patients not known to be at heightened risk of thrombotic events or to be allergic or intolerant to ASA. For those in whom ASA is contraindicated, prophylactic low molecular-weight heparin (LMWH)—such as enoxaparin 40 mg per day—should be used. For patients with a recent history of a thromboembolic event, full anticoagulation with LMWH is recommended, although warfarin could eventually be considered in patients with robust and stable platelet counts while on lenalidomide. Due to the low risk of venous thromboembolism associated with lenalidomide monotherapy (see New Directions, to be mentioned later), thromboprophylaxis in this scenario is not indicated.

Practice considerations are as follows.

- For lenalidomide monotherapy, the decision for thromboprophylaxis should be based on medical considerations. Some panel members felt strongly that thromboprophylaxis should be employed routinely in this setting.
- In the absence of contraindications, all patients on Len/Dex therapy should receive thromboprophylaxis. For patients without a history of thromboembolism or other known thrombotic conditions, ASA 81 or 325 mg per day is recommended. Prophylactic doses of LMWH (e.g., enoxaparin 40 mg sc daily) represent an alternative for such low-risk patients.
- Therapeutic anticoagulation with LMWH is recommended as thromboprophylaxis in patients with a recent history of thromboembolism or other known thrombotic disorder. Warfarin may be considered in patients with stable and reliable platelet counts over 100 × 10^9^/L.
- Thromboprophylaxis should be held if the platelet count drops below 50 × 10^9^/L and restarted when patients recover over that threshold.

#### 5.2.3. Rashes

Rashes occur in up to 29% of patients on the Len/Dex regimen [58]. These rashes occur most frequently during the first few weeks of treatment, are usually self-limited, and are severe in only a minority of patients. Nevertheless, Stevens-Johnson syndrome and toxic epidermal necrolysis have been reported and can be fatal. For localized rashes, antihistamines and topical steroids are usually sufficient. For mild but more extensive rashes, short-duration systemic low-dose steroids are usually needed. When rashes are more severe, dose interruption, reduction, or permanent discontinuation may be required, depending on clinical judgment. Importantly, patients with a past history of a severe rash associated with thalidomide should not receive lenalidomide. Of interest, one case of skin hypersensitivity reaction to lenalidomide with successful desensitization has been reported [59]. A similar case has been described for thalidomide [60], further supporting this intervention for those experiencing type I hypersensitivity to lenalidomide. Recommended management of rashes is summarized in Table 7.

Practice considerations are as follows.

- If a rash becomes severe, lenalidomide dose may be reduced, interrupted, or discontinued; otherwise, antihistamines and steroids are usually sufficient.

#### 5.2.4. Teratogenicity

Since lenalidomide could potentially be teratogenic in humans, precautions in females with child-bearing potential and males are important to avoid birth defects. In order to reduce these risks, the RevAid program provides a safe access to lenalidomide by stipulating a number of conditions for potential patients. For females of child-bearing potential, birth control using complete abstinence or two contraception methods is mandatory, beginning four weeks before initiation of lenalidomide and up to four weeks after. For males, complete abstinence or use of latex condoms during sexual contact with females of child-bearing potential is mandatory. While it is unknown whether lenalidomide is excreted in breast milk, breastfeeding is generally not recommended.

Practice considerations are as follows.

- Females of child-bearing potential and males in sexual contact with such females who are on Len/Dex treatment must use multiple contraception methods.

#### 5.2.5. Other

General symptoms such as fatigue and asthenia are reported at a similar frequency with Len/Dex as with dexamethasone monotherapy. However, these symptoms can become a reason for lenalidomide dose modification or discontinuation, especially in the elderly. Diarrhea and constipation have both been described, each occurring in approximately 20% of patients [45]. Although these symptoms can be routinely managed, our experience indicates that diarrhea may be particularly problematic in certain patients and that ongoing treatment with loperamide or similar agents may allow continuation of full doses of lenalidomide. The fact that lenalidomide capsules contain lactose might contribute to the gastrointestinal side effects noted in some patients.

For unexplained reasons, Len/Dex combination has been associated with a higher incidence of grade 3-4 atrial fibrillation compared to dexamethasone alone (4% versus 1.1%, resp.) [14]. Other side effects, such as loss of appetite and muscle cramps, may be bothersome to patients receiving treatment on a long-term basis. We have found that the use of quinine sulphate 200–300 mg per day is often effective in reducing the incidence and frequency of muscle cramps in significantly affected patients [61]. Anecdotally, patients with severe muscle cramps not completely controlled with quinine have derived relief from daily low doses of benzodiazepines such as clonazepam.

Tumor lysis syndrome has been described with lenalidomide, but it is more often a concern in chronic lymphocytic leukemia patients treated with this agent. Its occurrence in MM has not been well evaluated but appears to be uncommon. Nevertheless, tumor lysis syndrome can occur in any patient with a hematologic malignancy and a high tumor burden or with renal impairment. Thus, proper hydration and monitoring of electrolytes, creatinine, and uric acid is advisable in patients with a high tumor load and/or rapidly proliferating disease.

In contrast to that of thalidomide, the incidence of peripheral neuropathy with lenalidomide is very low [14]. Some cases of neurologic deterioration have been described with lenalidomide, but they might be due to the evolution of prior neuropathy. A recent observational study on the clinical course of peripheral neuropathy during lenalidomide treatment concluded that this therapy does not worsen peripheral neuropathy [62].

Practice considerations are as follows.

- Patients with significant diarrhea may require agents such as loperamide on a regular basis.
- Quinine sulphate 200–300 mg per day can reduce muscle cramps in affected patients.
- Although tumor lysis syndrome is considerably more common in patients treated with lenalidomide for chronic lymphocytic leukemia, myeloma patients with a high tumor load and/or rapidly proliferating disease may be at risk for this complication, especially if renal insufficiency is present. Proper hydration and laboratory monitoring is advisable in such patients when lenalidomide is initiated [63, 64].

### 5.3. Second Primary Malignancies

Emerging data from maintenance therapy studies using lenalidomide (IFM 05-02, CALGB 100104, and MM-015) suggest that long-term use of this agent might be associated with the development of second primary malignancies (SPM). However, in RRMM, after a median followup of 48 months for surviving patients, MM-009 and MM-010 have shown a low incidence of SPM. Furthermore, SPM rates were similar for patients on Len/Dex versus those on dexamethasone alone [40].

After an exhaustive review of clinical trials and post-marketing data, the European Medicines Agency issued a statement on September 23, 2011 to the effect that “the benefit-risk balance for lenalidomide remains positive within its approved patient population but advises doctors of the risk of new cancers as a result of treatment with the medicine.” This analysis found that there were 3.98 cases of new cancer for every 100 patient-years in patients receiving lenalidomide compared with 1.38 cases in those not receiving lenalidomide in the approved population (Press Release 23 Sept 2011, European Medicines Agency, http://www.ema.europa.eu/). These included skin cancers as well as hematologic malignancies and some invasive solid tumors. Health Canada issued a similar statement in May 2012, recommending careful evaluation of patients “before and during treatment in order to screen for the occurrence of new malignancies” (http://hc-sc.gc.ca/dhp-mps/medeff/advisories-avis/prof/_2012/revlimid_hpc-cps-eng.php, accessed July 2012).

The panel therefore reiterates that patients on lenalidomide should be watched for signs or symptoms of a new cancer. Routine cancer screening should be followed, per Canadian or local guidelines [65, 66]. 

Practice consideration are as follows.

- The efficacy of lenalidomide in RRMM outweighs the small risk of developing a secondary malignancy.
- Physicians and patients should be aware of this small risk; routine Canadian cancer screening measures should be performed, and any signs or symptoms of a possible second cancer should be evaluated and reported, if appropriate, to the RevAid program.

### 5.4. Monitoring of Adverse Events

Proper monitoring is required to note emerging side effects and to prevent potential treatment complications. A complete blood count with differential should be obtained every two weeks during the first 3 cycles and subsequently every month before a new cycle. Serum creatinine should be obtained before each cycle in order to adjust the lenalidomide dose according to impaired renal function. Because of possible liver toxicity [67] or thyroid dysfunction [68] associated with lenalidomide therapy, liver function tests including aspartate aminotransferase (AST), alanine aminotransferase (ALT), and bilirubin, as well as thyroid function tests should be done periodically throughout the treatment. Because atrial fibrillation remains a relatively rare event, serial electrocardiograms (ECGs) are not routinely required. For females of child-bearing potential, two pregnancy tests must be negative before starting lenalidomide: at 7–14 days and at 24 hours before administration of the drug. During the treatment, pregnancy tests should be conducted weekly for the first four weeks, then monthly (or every two weeks if menstrual cycles are irregular) until four weeks after treatment cessation.

### 5.5. New Directions

Given the established efficacy and favorable toxicity profile of lenalidomide in RRMM, this agent has now been evaluated at different time points in the disease course, as well as in combination with drugs other than dexamethasone alone (combination therapy) [20–29, 31–34, 68–75]. Combination of lenalidomide with alkylators, anthracyclines, and/or bortezomib yields very high remission rates (Table 8). So far, no randomized trials have established the superiority of a 3- or 4-drug combination over Len/Dex in terms of PFS or OS. Results of an induction trial, comparing Len/dex with MPT (MM020), are anticipated later this year.

In addition, even though lenalidomide—given with dexamethasone—is currently approved only for use after one prior therapy, there is considerable interest in employing this drug as part of initial therapy in newly diagnosed patients. Options in this setting include its administration in induction regimens in patients both eligible and ineligible for ASCT, in addition to its use as maintenance therapy after ASCT.

Phase III trials have now been initiated in these settings, and the available results are summarized in Table 9. Two recent randomized trials indicate that posttransplant maintenance therapy with single agent lenalidomide started 60–100 days after ASCT significantly improves PFS, and one of these trials has noted a survival advantage in the lenalidomide arm [27]. Adoption of lenalidomide maintenance as a standard of care will depend on the identification of the subgroups most likely to benefit, the risk of late complications such as SPM, and the cost implications of such a strategy. On balance, it is likely that the results of recent/ongoing randomized studies will lead to expanded applications of lenalidomide in the treatment of patients with MM.

## 6. Conclusions

Based on available evidence, Len/Dex appears to be an effective and safe treatment strategy for RRMM patients, regardless of the type and number of prior therapies. In order to ensure optimal balance between efficacy and tolerability, lenalidomide dose and schedule should be adjusted based on creatinine clearance and presence of neutropenia and thrombocytopenia; dexamethasone should typically be administered at weekly doses of 20–40 mg, and treatment should be continued until disease progression or toxicity, even in patients requiring dose reduction.

Although certain adverse events can occur with Len/Dex, the following precautions can significantly reduce their impact: (1) Lenalidomide interruption and dose modification should follow established guidelines, with judicious use of G-CSF and transfusions if needed to avoid potential hematological toxicities; (2) all patients should receive thromboprophylaxis unless contraindicated. In most patients without a history of thrombosis, 81 mg of ASA is sufficient; alternatively, prophylactic doses of LMWH may be administered. Patients with a recent history of thromboembolism or known thrombotic disorder require full anticoagulation while on Len/Dex, usually consisting of LMWH; patients with stable platelet counts over 100 × 109/L can be considered for coumadin; (3) ESAs should be used cautiously, and if this treatment is used, the hemoglobin target should be <120 g/L; (4) females of child-bearing potential and males in sexual contact with such females must use multiple contraception methods.

Future studies are needed to elucidate the role of lenalidomide as part of initial MM therapy, as well as maintenance therapy after ASCT. Also, while various three- and four-drug combinations including lenalidomide as the backbone appear promising, not enough information is available to recommend combination treatment outside of a clinical trial.

## Figures and Tables

**Table 1:** Dose adjustments at the start of therapy according to renal function [14].

Renal function	Dose*
Mild renal impairment ( 60 ≤ CrCl < 90 mL/min)	25 mg (normal dose) every 24 hours
Moderate renal impairment (30 ≤ CrCl < 60 mL/min)	10 mg^†^ every 24 hours
Severe renal impairment (CrCl < 30 mL/min, not requiring dialysis)	15 mg every 48 hours
End-stage renal disease (CrCl < 30 mL/min, requiring dialysis)	5 mg once daily. On dialysis days, the dose should be administered following dialysis

*While maintaining a treatment cycle of 21 out of 28 days.

^†^Dose may be escalated to 15 mg once daily after two cycles if patient does not respond to and is tolerating treatment.

CrCl: creatinine clearance.

**Table 2:** Lenalidomide dose reduction levels with adequate renal function.

Dose level	Lenalidomide dose (mg)
Initial dose	25
First reduction level	15
Second reduction level	10
Third reduction level	5
Fourth reduction level	Discontinuation

**Table 3:** Lenalidomide dose adjustment for neutropenia.

Neutrophil count	Recommendations
<1 × 10^9^/L on day 1 of a cycle	Delay start of the cycle for a week, until neutrophil count ≥1 × 10^9^/L

<1 × 10^9^/L during a cycle	Interruption of lenalidomide until next cycle (dexamethasone should be continued)

Returning to ≥1 × 10^9^/L on next cycle	Continue lenalidomide at same dose ± addition of G-CSF, if no other significant toxicities needing dose reduction Reduce lenalidomide to the first reduction level if other significant toxicities observed

For each subsequent drop <1 × 10^9^/L	Interrupt lenalidomide treatment

Returning to ≥1 × 10^9^/L on next cycle	Resume lenalidomide at next dose reduction level

G-CSF: granulocyte-colony stimulating factor.

**Table 4:** Lenalidomide dose adjustment for thrombocytopenia.

Platelet count	Recommendations
<30 × 10^9^/L on day 1 of a cycle	Delay start of the cycle for a week, until platelet count ≥30 × 10^9^/L
<30 × 10^9^/L during a cycle	Interruption of lenalidomide until next cycle (dexamethasone should be continued)
Returning to ≥30 × 10^9^/L on next cycle	Reduce lenalidomide to the first reduction level
For each subsequent drop <30 × 10^9^/L	Interrupt lenalidomide treatment
Returning to ≥30 × 10^9^/L on next cycle	Resume lenalidomide at next dose reduction level

**Table 5:** Adverse prognostic factors identified by multivariate analysis in patients with relapsed/refractory myeloma treated with lenalidomide and dexamethasone.

Reference	Study population	PFS/TTP	Overall survival
Reece et al., 2009 [15]	130 RRMM patients treated with Len/Dex	Del(17p13) Elevated creatinine Prior bortezomib Prior thalidomide	Del(17p13) Elevated creatinine Prior bortezomib Prior thalidomide Age >65 yrs

Klein et al., 2011 [16]	92 RRMM patients treated with Len/Dex	Del(13q) if associated with other abnormalities	Del(17p13) Amp(1q21)

Avet-Loiseau et al., 2010 [17]	207 “heavily pretreated” RRMM patients treated with Len/Dex	Progression during thalidomide Hemoglobin <100 Del 13q	Progression during thalidomide

Dimopoulos et al., 2010 [18]	99 RRMM patients treated with Len/Dex (*n* = 50) or Len/Dex + bortezomib	t(4;14) Del(17p13) Thalidomide resistance Elevated LDH Extramedullary disease	Del(13q)
			Amp(1q21)
			Del(17p13)
			Thalidomide resistance ISS
			Bortezomib resistance
			Elevated LDH
			Extramedullary disease

PFS: progression-free survival; TTP: time to progression; RRMM: relapsed or refractory multiple myeloma; Len/Dex: lenalidomide combined with dexamethasone; LDH: lactate dehydrogenase; ISS: international staging system.

**Table 6:** The effect of Len/Dex treatment according to prior response to thalidomide. Adapted from Wang et al. [19].

	Thalidomide sensitive^1^			Thalidomide relapsed^2^			Thalidomide resistant^3^		
	Len/Dex	Placebo/Dex	*P*	Len/Dex	Placebo/Dex	*P*	Len/Dex	Placebo/Dex	*P*
Overall response rates (PR or better) %	64.8	17.1	<0.001	41.9	5.9	<0.01	50	20.8	0.042

Response duration, mo (95% CI)	13.4 (7.0 to NE)	3.2 (2.3 to NE)	0.009	8.8 (5.3 to NE)	NE (8.6 to NE)	0.77	NE (6.0 to NE)	NE (6.0 to NE)	0.22

Median PFS, mo (95% CI)	9.3 (5.6 to 18.0)	4.6 (3.9 to 4.7)	<0.001	7.8 (5.2 to 11.1)	3.7 (2.8 to 6.5)	0.002	7.0 (4.9 to 16.9)	3.7 (2.1 to 8.4)	0.013

^1^Sensitive: patients with stable disease or better who did not progress while on thalidomide.

^2^Relapsed: patients with stable disease or better who progressed while on thalidomide.

^3^Resistant: patients who progressed on thalidomide but never responded to thalidomide.

Len/Dex: lenalidomide combined with dexamethasone; PR: partial response; PFS: progression-free survival; NE: not estimable.

**Table 7:** Management of rashes due to lenalidomide.

Signs/symptoms	Treatment
Localized maculopapular rash	Topical steroids; antihistamines
Widespread maculopapular rash	Hold lenalidomide; topical or oral steroids depending on severity; antihistamines; after resolution, restart lenalidomide at lower dose
Generalize erythroderma or desquamation	Hold lenalidomide; oral steroids; Dermatology consultation; do not restart lenalidomide
Urticaria	Hold lenalidomide; symptomatic management with antihistamines ± oral steroids; after resolution, may attempt desensitization if reinitiation of lenalidomide is planned

**Table 8:** Summary of emerging lenalidomide combination therapies in the first- and second-line treatment of multiple myeloma.

Combination	First line		≥Second line	
	Efficacy	Major toxicities	Efficacy	Major toxicities
MPR Palumbo et al., 2007 [20]	81% ≥ PR	Hematological toxicity		

MPR-R Palumbo et al., 2010 [21]			75% ≥ PR	Hematological toxicity, infections

RMPT Palumbo et al., 2010 [22]				

RVD Richardson et al., 2009, 2010 [23, 24]	100% ≥ PR	Hematological toxicity, sensory neuropathy	61% ≥ MR	Hematological toxicity

CPR Reece et al., 2010 [25]			94% ≥ MR	Hematological toxicity

CRD Schey et al., 2010 [26]			81% ≥ PR	Hematological toxicity

RVDC Kumar et al., 2010 [27]	96% ≥ PR	Hematological toxicity, sensory neuropathy

CRd Kumar et al., 2011 [28]	85% ≥ PR	Hematological toxicity

RVDD Jakubowiak et al., 2011 [29]	95% ≥ PR	Fatigue, constipation, sensory neuropathy, infection

MPR: melphalan, prednisone, lenalidomide; PR: partial response; MPR-R: MPR + lenalidomide maintenance until progression; RMPT: lenalidomide, melphalan, prednisone, thalidomide; RVD: lenalidomide, bortezomib, dexamethasone; MR: minimal response; CPR: cyclophosphamide, prednisone, lenalidomide; CRD: cyclophosphamide, lenalidomide, dexamethasone; RVDC: lenalidomide, bortezomib, dexamethasone, cyclophosphamide; CRd: cyclophosphamide, lenalidomide, dexamethasone; RVDD: lenalidomide, bortezomib, pegylated liposomal doxorubicin, dexamethasone.

**Table 9:** Summary of phase III trials evaluating new indications for lenalidomide in the treatment of multiple myeloma.

New indications	Trials	Regimens	Response rate	PFS	OS
	ECOG E4A03 Rajkumar et al., 2010 [30]	Len + HD dex Len + LD dex	79% 68%	19.1 mos 25.3 mos	75% (2-yr) 87% (2-yr)
					
	MM-015 Palumbo et al. 2012 [31] *N* = 348 (Age 65–75)	MP	47%	12 mos	65% (3 yrs)
		MPR		15 mos	~70% (3 yrs)
		MPR-R	79%	31 mos	73% (3 yrs)
Induction therapy	MM-020	MPT Len + LD dex until progression Len + LD dex for 18 mos	In progress	In progress	In progress
	Palumbo et al., 2011 [32] *N* = 402	Len + LD dex × 4 cycles→MPR	20%	54% (2 yrs)	87% (2 yrs)
		Len + LD dex × 4 cycles→ASCT × 2	25%	73% (2 yrs)	90% (2 yrs)

Maintenance therapy after ASCT	IFM2005-02 Attal et al. 2010 [33] *N* = 614	Len Placebo	— —	42 mos 24 mos	81% (3 yrs) 81% (3 yrs)
	CALGB 100104 McCarthy et al. 2010 [34] *N* = 568	Len Placebo	— —	43.6 mos 21.5 mos	~80% (3 yrs) ~80% (3 yrs)

Induction and maintenance ± ASCT in newly diagnosed patients	IFM/Dana Farber trial	VRD × 8→Len maintenance × 1 yr (ASCT at progression) VRD × 3 →ASCT→Len maintenance × 1 yr	In progress	In progress	In progress

CR: compete response; PFS: progression-free survival; OS: overall survival; Len: lenalidomide; HD dex: high-dose dexamethasone; LD dex: low-dose dexamethasone; MP: melphalan, prednisone; MPR: melphalan, prednisone, lenalidomide; MPR-R: MPR + lenalidomide maintenance until progression; MPT: melphalan, prednisone, thalidomide; ASCT: autologous stem cell transplantation; VRD: bortezomib, lenalidomide, dexamethasone.

## References

[1] Canadian Cancer Society's Steering Committee on Cancer Statistics (2011). *Canadian Cancer Statistics*.

[2] Kumar SK, Rajkumar SV, Dispenzieri A (2008). Improved survival in multiple myeloma and the impact of novel therapies. *Blood*.

[3] Venner CP, Connors JM, Sutherland HJ (2011). Novel agents improve survival of transplant patients with multiple myeloma including those with high-risk disease defined by early relapse (<12 months). *Leukemia and Lymphoma*.

[4] Richardson P, Jagannath S, Hussein M (2009). Safety and efficacy of single-agent lenalidomide in patients with relapsed and refractory multiple myeloma. *Blood*.

[5] Gandhi AK, Kang J, Capone L (2010). Dexamethasone synergizes with lenalidomide to inhibit multiple myeloma tumor growth, but reduces lenalidomide-induced immunomodulation of T and NK cell function. *Current Cancer Drug Targets*.

[6] Mitsiades N, Mitsiades CS, Poulaki V (2002). Apoptotic signaling induced by immunomodulatory thalidomide analogs in human multiple myeloma cells: therapeutic implications. *Blood*.

[7] Hideshima T, Chauhan D, Shima Y (2000). Thalidomide and its analogs overcome drug resistance of human multiple myeloma cells to conventional therapy. *Blood*.

[8] Verhelle D, Corral LG, Wong K (2007). Lenalidomide and CC-4047 inhibit the proliferation of malignant B cells while expanding normal CD34^+^ progenitor cells. *Cancer Research*.

[9] De Luisi A, Ferrucci A, Coluccia AML (2011). Lenalidomide restrains motility and overangiogenic potential of bone marrow endothelial cells in patients with active multiple myeloma. *Clinical Cancer Research*.

[10] Kotla V, Goel S, Nischal S (2009). Mechanism of action of lenalidomide in hematological malignancies. *Journal of Hematology and Oncology*.

[11] Davies F, Baz R (2010). Lenalidomide mode of action: Linking bench and clinical findings. *Blood Reviews*.

[12] Weber DM, Chen C, Niesvizky R (2007). Lenalidomide plus dexamethasone for relapsed multiple myeloma in North America. *The New England Journal of Medicine*.

[13] Dimopoulos M, Spencer A, Attal M (2007). Lenalidomide plus dexamethasone for relapsed or refractory multiple myeloma. *The New England Journal of Medicine*.

[15] Reece D, Song KW, Fu T (2009). Influence of cytogenetics in patients with relapsed or refractory multiple myeloma treated with lenalidomide plus dexamethasone: adverse effect of deletion 17p13. *Blood*.

[16] Klein U, Jauch A, Hielscher T (2011). Chromosomal aberrations ^+^1q21 and del(17p13) predict survival in patients with recurrent multiple myeloma treated with lenalidomide and dexamethasone. *Cancer*.

[17] Avet-Loiseau H, Soulier J, Fermand JP (2010). Impact of high-risk cytogenetics and prior therapy on outcomes in patients with advanced relapsed or refractory multiple myeloma treated with lenalidomide plus dexaméthasone. *Leukemia*.

[18] Dimopoulos MA, Kastritis E, Christoulas D (2010). Treatment of patients with relapsed/refractory multiple myeloma with lenalidomide and dexamethasone with or without bortezomib: prospective evaluation of the impact of cytogenetic abnormalities and of previous therapies. *Leukemia*.

[19] Wang M, Dimopoulos MA, Chen C (2008). Lenalidomide plus dexamethasone is more effective than dexamethasone alone in patients with relapsed or refractory multiple myeloma regardless of prior thalidomide exposure. *Blood*.

[20] Palumbo A, Falco P, Corradini P (2007). Melphalan, prednisone, and lenalidomide treatment for newly diagnosed myeloma: a report from the GIMEMA—Italian Multiple Myeloma Network. *Journal of Clinical Oncology*.

[21] Palumbo A, Delforge M, Catalano J (2010). A phase 3 study evaluating the efficacy and safety of lenalidomide combined with melphalan and prednisone in patients >= 65 years with newly diagnosed multiple myeloma (ndmm): continuous use of lenalidomide vs fixed-duration regimens. *Blood*.

[22] Palumbo A, Larocca A, Falco P (2010). Lenalidomide, melphalan, prednisone and thalidomide (RMPT) for relapsed/refractory multiple myeloma. *Leukemia*.

[23] Richardson PG, Weller E, Jagannath S (2009). Multicenter, phase I, dose-escalation trial of lenalidomide plus bortezomib for relapsed and relapsed/refractory multiple myeloma. *Journal of Clinical Oncology*.

[24] Richardson PG, Weller E, Lonial S (2010). Lenalidomide, bortezomib, and dexamethasone combination therapy in patients with newly diagnosed multiple myeloma. *Blood*.

[25] Reece DE, Masih-Khan E, Khan A (2010). Phase I-II trial of oral cyclophosphamide, prednisone and lenalidomide (Revlimid) for the treatment of patients with relapsed and refractory multiple myeloma. *Blood*.

[26] Schey SA, Morgan GJ, Ramasamy K (2010). The addition of cyclophosphamide to lenalidomide and dexamethasone in multiply relapsed/refractory myeloma patients; a phase I/II study. *British Journal of Haematology*.

[27] Kumar SK, Flinn I, Noga SJ (2010). Bortezomib, dexamethasone, cyclophosphamide and lenalidomide combination for newly diagnosed multiple myeloma: phase 1 results from the multicenter EVOLUTION study. *Leukemia*.

[28] Kumar SK, Lacy MQ, Hayman SR (2011). Lenalidomide, cyclophosphamide and dexamethasone (CRd) for newly diagnosed multiple myeloma: results from a phase 2 trial. *American Journal of Hematology*.

[29] Jakubowiak AJ, Griffith KA, Reece DE (2011). Lenalidomide, bortezomib, pegylated liposomal doxorubicin, and dexamethasone in newly diagnosed multiple myeloma: a phase 1/2 multiple myeloma research consortium trial. *Blood*.

[30] Rajkumar SV, Jacobus S, Callander NS (2010). Lenalidomide plus high-dose dexamethasone versus lenalidomide plus low-dose dexamethasone as initial therapy for newly diagnosed multiple myeloma: an open-label randomised controlled trial. *The Lancet Oncology*.

[31] Palumbo A, Hajek R, Delforge M (2012). Continuous lenalidomide treatment for newly diagnosed multiple myeloma. *Blood*.

[32] Palumbo A, Cavallo F, Hardan i (2011). Melphalan/Prednisone/Lenalidomide (MPR) Versus High-Dose Melphalan and Autologous Transplantation (MEL200) in Newly Diagnosed Multiple Myeloma (MM) Patients <65 Years: results of a randomized phase III study. *Blood*.

[33] Attal M, Cances-Lauwers V, Marit G (2010). Lenalidomide maintenance treatment after stem-cell transplantation for multiple myeloma. *The New England Journal of Medicine*.

[34] McCarthy P, Owzar K, Anderson K (2010). Phase III Intergroup study of lenalidomide versus placebo maintenance therapy following single autologous hematopoietic stem cell transplantation (AHSC T) for multiple myeloma: CALGB, 100104. *Blood*.

[35] Stadtmauer EA, Weber DM, Niesvizky R (2009). Lenalidomide in combination with dexamethasone at first relapse in comparison with its use as later salvage therapy in relapsed or refractory multiple myeloma. *European Journal of Haematology*.

[36] Kleber M, Ihorst G, Deschler B (2009). Detection of renal impairment as one specific comorbidity factor in multiple myeloma: multicenter study in 198 consecutive patients. *European Journal of Haematology*.

[37] San Miguel JF, Dimopoulos M, Weber D (2007). Dexamethasone dose adjustments seem to result in better efficacy and improved tolerability in patients with relapsed/refractory multiple myeloma who are treated with lenalidomide/dexamethasone (MM-009/010 sub-analysis). *Blood*.

[38] Hsu AK, Quach H, Tai T (2011). The immunostimulatory effect of lenalidomide on NK-cell function is profoundly inhibited by concurrent dexamethasone therapy. *Blood*.

[39] San-Miguel JF, Dimopoulos MA, Stadtmauer EA (2011). Effects of lenalidomide and dexamethasone treatment duration on survival in patients with relapsed or refractory multiple myeloma treated with lenalidomide and dexamethasone. *Clinical Lymphoma, Myeloma and Leukemia*.

[40] Dimopoulos M, Orlowski NR (2011). Lenalidomide and dexamethasone (LEN plus DEX) treatment in relapsed/refractory multiple myeloma (RRMM) patients (pts) and risk of second primary malignancies (SPM): analysis of MM-009/010.. *Journal of Clinical Oncology*.

[41] Nair B, Van Rhee F, Shaughnessy JD (2010). Superior results of total therapy 3 (2003-33) in gene expression profiling-defined low-risk multiple myeloma confirmed in subsequent trial 2006-66 with VRD maintenance. *Blood*.

[42] Guglielmelli T, Bringhen S, Rrodhe S (2011). Previous thalidomide therapy may not affect lenalidomide response and outcome in relapse or refractory multiple myeloma patients. *European Journal of Cancer*.

[43] Madan S, Lacy MQ, Dispenzieri A (2011). Efficacy of retreatment with immunomodulatory drugs (IMiDs) in patients receiving IMiDs for initial therapy of newly diagnosed multiple myeloma. *Blood*.

[44] Altekruse SF KC, Krapcho M, Neyman N (2010). *SEER Cancer Statistics Review, 1975–2007*.

[45] Chen C, Reece DE, Siegel D (2009). Expanded safety experience with lenalidomide plus dexamethasone in relapsed or refractory multiple myeloma. *British Journal of Haematology*.

[46] Ishak JD, Weber MA, Knight D (2008). Declining rates of adverse events and dose modifications with lenalidomide in combination with dexamethasone. *Blood*.

[47] Lonial SB, Swern R, Weber AS (2009). Neutropenia is a predictable and early event in affected patients with relapsed/refractory multiple myeloma treated with lenalidomide in combination with dexamethasone. *Blood*.

[48] Niesvizky R, Naib T, Christos PJ (2007). Lenalidomide-induced myelosuppression is associated with renal dysfunction: adverse events evaluation of treatment-naïve patients undergoing front-line lenalidomide and dexamethasone therapy. *British Journal of Haematology*.

[49] Kumar S, Dispenzieri A, Lacy MQ (2007). Impact of lenalidomide therapy on stem cell mobilization and engraftment post-peripheral blood stem cell transplantation in patients with newly diagnosed myeloma. *Leukemia*.

[50] Popat U, Saliba R, Thandi R (2009). Impairment of filgrastim-induced stem cell mobilization after prior lenalidomide in patients with multiple myeloma. *Biology of Blood and Marrow Transplantation*.

[51] Nazha A, Cook R, Vogl DT (2011). Stem cell collection in patients with multiple myeloma: impact of induction therapy and mobilization regimen. *Bone Marrow Transplantation*.

[52] Mark T, Stern J, Furst JR (2008). Stem cell mobilization with cyclophosphamide overcomes the suppressive effect of lenalidomide therapy on stem cell collection in multiple myeloma. *Biology of Blood and Marrow Transplantation*.

[53] Micallef INM, Ho AD, Klein LM, Marulkar S, Gandhi PJ, McSweeney PA (2011). Plerixafor (Mozobil) for stem cell mobilization in patients with multiple myeloma previously treated with lenalidomide. *Bone Marrow Transplantation*.

[54] Baz RL, Hussein S, Swern M (2010). Lenalidomide (LEN) therapy in combination with dexamethasone (DEX) is associated with a low incidence of viral infections. *Blood*.

[55] Carrier M, Le Gal G, Tay J, Wu C, Lee AY (2011). Rates of venous thromboembolism in multiple myeloma patients undergoing immunomodulatory therapy with thalidomide or lenalidomide: a systematic review and meta-analysis. *Journal of Thrombosis and Haemostasis*.

[56] Palumbo A, Cavo M, Bringhen S (2011). Aspirin, warfarin, or enoxaparin thromboprophylaxis in patients with multiple myeloma treated with thalidomide: a phase III, open-label, randomized trial. *Journal of Clinical Oncology*.

[57] Larocca A, Cavallo F, Bringhen S (2012). Aspirin or enoxaparin thromboprophylaxis for newly-diagnosed multiple 11 myeloma patients treated with lenalidomide. *Blood*.

[58] Sviggum HP, Davis MDP, Rajkumar SV, Dispenzieri A (2006). Dermatologic adverse effects of lenalidomide therapy for amyloidosis and multiple myeloma. *Archives of Dermatology*.

[59] Phillips J, Kujawa J, Davis-Lorton M, Hindenburg A (2007). Successful desensitization in a patient with lenalidomide hypersensitivity. *American Journal of Hematology*.

[60] Nucera E, Schiavino D, Hohaus S (2008). Desensitization to thalidomide in a patient with multiple myeloma. *Clinical Lymphoma and Myeloma*.

[61] El-Tawil S, Al Musa T, Valli H, Lunn MP, El-Tawil T, Weber M (2010). Quinine for muscle cramps. *Cochrane database of systematic reviews*.

[62] Zambello R, Berno T, Candiotto L (2011). Peripherial neuropathy clinical course during lenalidomide therapy for relapsed/refractory multiple myeloma: a single-centre prospective non interventional study. *Haematologica*.

[63] Wendtner C-M, Hillmen P, Mahadevan D (2012). Final results of a multicenter phase 1 study of lenalidomide in patients with relapsed or refractory chronic lymphocytic leukemia. *Leukemia and Lymphoma*.

[64] Chim CS (2008). Rapid complete remission in multiple myeloma with bortezomib/thalidomide/ dexamethasone combination therapy following development of tumor lysis syndrome. *Cancer Chemotherapy and Pharmacology*.

[65] Leddin DJ, Enns R, Hilsden R (2010). Canadian Association of Gastroenterology position statement on screening individuals at average risk for developing colorectal cancer: 2010. *Canadian Journal of Gastroenterology*.

[66] Izawa J, Siemens KLD (2010). Prostate cancer screening: Canadian guidelines 2011. *Canadian Urological Association Journal*.

[67] Hussain S, Browne R, Chen J, Parekh S (2007). Lenalidomide-induced severe hepatotoxicity. *Blood*.

[68] Figaro MK, Clayton W, Usoh C (2011). Thyroid abnormalities in patients treated with lenalidomide for hematological malignancies: results of a retrospective case review. *American Journal of Hematology*.

[69] Gay F, Vincent Rajkumar SS, Falco P (2010). Lenalidomide plus dexamethasone vs. lenalidomide plus melphalan and prednisone: a retrospective study in newly diagnosed elderly myeloma. *European Journal of Haematology*.

[70] Jakubowiak AJ, Dytfeld D, Jagannath S (2010). Carfilzomib, lenalidomide, and dexamethasone in newly diagnosed multiple myeloma: initial results of phase I/II MMRC trial. *Blood*.

[71] Lentzsch S, 'Sullivan AO, Kennedy R (2010). Combination of bendamustine, lenalidomide, and dexamethasone in patients with refractory or relapsed multiple myeloma is safe and highly effective: results of a phase i clinical trial. *Blood*.

[72] Lonial S, Vij R, Harousseau JL (2010). Elotuzumab in combination with lenalidomide and low-dose dexamethasone in patients with relapsed/refractory multiple myeloma: interim results of a phase 1 study. *Blood*.

[73] Palumbo A, Falco P, Falcone A (2009). Melphalan, prednisone, and lenalidomide for newly diagnosed myeloma: kinetics of neutropenia and thrombocytopenia and time-to-event results. *Clinical Lymphoma and Myeloma*.

[74] White DJ, Bahlis NJ, Marcellus DC (2008). Phase II testing of lenalidomide plus melphalan for previously untreated older patients with multiple myeloma: the NCIC CTG MY. 11 trial. *Blood*.

[75] Bensinger W, Wang M, Orlowski RZ (2010). Dose-escalation study of carfilzomib (CFZ) plus lenalidomide (LEN) plus low-dose dexamethasone (Dex) (CRd) in relapsed/refractory multiple myeloma (R/R MM). *Journal of Clinical Oncology*.

[76] Chen C, Baldassarre F, Kanjeekal S (2012). *Lenalidomide in Multiple Myeloma*.

